# Advancing Hepatitis C Elimination through Opt-Out Universal Screening and Treatment in Carceral Settings, United States

**DOI:** 10.3201/eid3013.230859

**Published:** 2024-04

**Authors:** Maeve McNamara, Nathan Furukawa, Emily J. Cartwright

**Affiliations:** Emory University, Atlanta, Georgia, USA (M. McNamara, E.J. Cartwright); Centers for Disease Control and Prevention, Atlanta (N. Furukawa, E.J. Cartwright);; Veterans Affairs Atlanta Health Care System, Decatur, Georgia, USA (E.J. Cartwright)

**Keywords:** hepatitis C virus, incarceration, opt-out universal screening, direct-acting antivirals, substance use treatment, viruses, United States

## Abstract

Incarcerated persons are infected with hepatitis C virus (HCV) at rates ≈10 times higher than that of the general population in the United States. To achieve national hepatitis C elimination goals, the diagnosis and treatment of hepatitis C in incarcerated persons must be prioritized. In 2022, the Centers for Disease Control and Prevention recommended that all persons receive opt-out HCV screening upon entry into a carceral setting. We review recommendations, treatments, and policy strategies used to promote HCV opt-out universal HCV screening and treatment in incarcerated populations in the United States. Treatment of hepatitis C in carceral settings has increased but varies by jurisdiction and is not sufficient to achieve HCV elimination. Strengthening universal HCV screening and treatment of HCV-infected incarcerated persons is necessary for HCV elimination nationwide.

Hepatitis C virus (HCV) infection is the most commonly reported bloodborne infection in the United States; the estimated prevalence was 2.2 million cases during 2017–2020 (*1*). According to 2023 estimates, HCV infection prevalence among incarcerated persons was ≈10 times that of the general US population (*2*). The United States Bureau of Justice Statistics estimated that >5 million persons were under the supervision of US adult carceral systems in 2020 (*3*,*4*). Cumulatively, ≈600,000 persons were released from state and federal prisons in 2020, and another 9 million persons cycled through local jails (*3*,*4*). Black men are 4.8 times more likely and Latino men are 1.3 times more likely to be incarcerated than White men in US prisons (*5*).

Injection drug use and, to a lesser degree, tattooing are the primary risk factors for HCV transmission during incarceration (*6*). Partly because of drug use criminalization, persons who inject drugs experience high rates of incarceration (*7*). Many persons are infected with HCV before incarceration, and continued injection drug use during incarceration is common. Tattooing rates during incarceration have been reported to be 8.7%–19.3% in the United States (*6*). Taken together, nonsterile injection practices during incarceration create opportunities for HCV infection and reinfection (*8*). Furthermore, cycles of reincarceration compound the risk for continued HCV transmission between previously incarcerated and nonincarcerated persons (*8*).

Left untreated, HCV can cause cirrhosis, liver cancer, and death; 13,895 deaths were attributed to HCV in the United States in 2021 (*9*). HCV infection alone contributes to a 61% increased risk for 2-year mortality among incarcerated persons (*10*). Fortunately, hepatitis C is curable in >95% of cases by using specific direct-acting antiviral (DAA) medications, approved by the Food and Drug Administration beginning in 2012 (*11*). Treatment can prevent liver damage, liver failure, and cancer; furthermore, DAA treatment can prevent ongoing HCV transmission (*12*–*17*). However, inequities exist in accessing DAA medications; DAA treatment is 30% less likely to be initiated among insured non-Hispanic Black persons than among non-Hispanic White persons (*18*). Furthermore, non-Hispanic Black persons are 3.2 times more likely and American Indian/Alaskan Native persons are 1.8 times more likely to die from HCV infection sequelae than non-Hispanic White persons (*9*).

We review policy strategies to implement HCV opt-out universal screening and treatment in incarcerated populations. Strengthening HCV elimination policies and practices in carceral settings is critical to achieving national HCV elimination.

## Hepatitis C Screening Evolution and Current Recommendations

Guidelines for testing and screening for HCV in the United States have evolved since the original recommendations were first published by the Centers for Disease Control and Prevention (CDC) in 1991 (Figure). Although risk-based HCV testing was recommended in 2003, it missed a substantial proportion of persons with HCV (*19*). During 2019–2020, the American Association for the Study of Liver Diseases (AASLD), Infectious Diseases Society of America (IDSA), US Preventive Services Task Force, and CDC recommended universal HCV screening for all adults at least once during a lifetime (*15*,*20*,*21*). CDC (2022) and AASLD/IDSA (2023) recommended universal opt-out HCV intake screening of incarcerated and detained persons (*22*,*23*).

**Figure:**
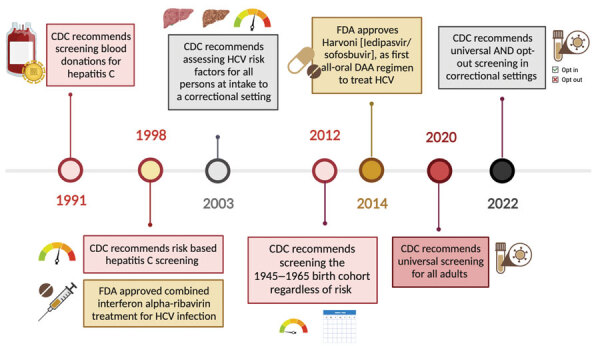
Timeline of hepatitis C virus screening recommendations and treatments that advance hepatitis C elimination in carceral settings, United States. Colored circles on the timeline indicate the year certain recommendations were made or hepatitis C treatments were approved. Other symbols are pictorial representations. CDC, Centers for Disease Control and Prevention; DAA, direct-acting antiviral; FDA, Food and Drug Administration; HCV, hepatitis C virus.

Models estimate that universal opt-out screening in US prisons would diagnose >122,000 HCV infections and prevent ≈13,000 new prison-associated infections, ≈2–3 times more than would be possible with risk-factor based assessments (*24*). Implementing universal opt-out HCV screening and associated treatment costs would increase state prison healthcare budgets by an estimated 12.4% (*24*). Thus, budgetary constraints might limit the broader adoption of universal opt-out HCV screening and treatment.

## HCV Screening in Carceral Settings: Real-World Examples

During 2004–2012, the Pennsylvania Department of Corrections (DOC) successfully began a universal opt-out screening program resulting in 93% of incarcerated persons screened for HCV at intake (*19*). Similarly, the Washington DOC successfully screened 83% of its incarcerated population during 2012–2016 (*25*). The Pennsylvania and Washington screening programs identified 18%–20% HCV seropositivity rates (Table 1) (*19*,*25*).

**Table 1:** Real-world examples of opt-out screening for hepatitis C virus in prisons and jails used to advance hepatitis C elimination in carceral settings, United States*

Reference	Institution	Years	Population	Policy	Outcome
(*19*)†	Pennsylvania state prisons	2004–2012	101,727 persons entering state prison	Universal opt-out testing at intake; diagnostic testing offered to persons with positive screening tests and subsequent evaluation for HCV therapy (only seropositivity rates reported).	A total of 101,727 persons were tested for HCV; of those, 18,454 (18.1%) were HCV positive.
(*25*)	Washington state prisons	2012–2016	24,567 persons entering state prison	Universal opt-out, laboratory-based HCV testing	A total of 24,567 (83%) persons were screened for HCV; of those, 4,921 (20%) were HCV positive. Of the 4,921 HCV-positive patients, 2,403 (49%) had hepatitis C virus RNA testing; 1,727 of 2,403 (72%) had HCV viremia.
(*51*)‡	NYC jails	2014–2017	121,371 persons with >1 admission to the NYC jail system	Opt-out HCV testing for a subset of jail entrants	A total of 40,219 (33%) persons were tested for HCV; of those, 4,665 (12%) were positive for HCV viremia and 248 (5%) were treated.
(*52*)	Durham County, NC, jail in collaboration with Durham Department of Public Health	Dec 2012–Mar 2014	669 persons entering local jail (5.6% of all entrants)	Opt-out HCV testing for a subset of jail entrants	A total of 669 (5.6%) persons were tested for HCV; of those, 88 (13.2%) were HCV positive. Of those 88 patients, 81 (92.0%) were tested for HCV RNA; 66 of 81 (81.5%) had HCV viremia. Of the 66 with viremia, 18 (27.3%) were referred to post-release medical care, 10 (55.6%) of whom attended their first appointment.

*HCV positive refers to a positive or reactive test for HCV antibodies, indicating current or prior exposure to HCV. Viremia is defined as a positive serum HCV RNA test. HCV, hepatitis C virus; NYC, New York, NY. †Does not include any data on RNA testing or HCV viremia. ‡Excluded persons who completed DAA treatment, started DAA treatment in the community, or who did not complete medical intake; this study only includes data for RNA testing and HCV viremia and excludes information on HCV antibody testing.

Partnerships between carceral facilities and departments of health are promising strategies to enact universal opt-out HCV testing. The Indiana Department of Health embedded an epidemiologist in the Indiana DOC and began universal HCV screening at intake, which ultimately identified a 12% intake viremia prevalence (*26*). The collaboration between Indiana’s Department of Health and DOC resulted in a transition to universal treatment, the creation of a peer education program, a community care transition program, and development of data tracking capabilities to generate HCV care cascades (*26*).

## Hepatitis C Treatment Evolution and Current Recommendations

During 1998–2014, interferon-based therapies were the gold standard for hepatitis C treatment but were ineffective, poorly tolerated, and unsafe for many persons (*27*). The approval of sofosbuvir in 2013 shifted the treatment paradigm toward safe, highly efficacious, oral DAA therapies that had >95% sustained virologic response (SVR) rates and few contraindications (*15*,*28*). SVR is defined as no detectable HCV RNA in blood after completing treatment. Attaining SVR after treatment with DAAs reduced all-cause mortality, end-stage liver disease, and hepatocellular carcinoma among Medicare beneficiaries during 2014–2016 (*14*). In 2019, the AASLD/IDSA recommended treating all patients with current HCV infection except those who had a short life expectancy and cannot be remediated by HCV therapy (*15*). Although treatment remains expensive, manufacturer competition and negotiated pricing have substantially driven down DAA costs.

Considerable costs associated with chronic liver disease can be prevented by treating HCV infection. In 2019, the estimated annual cost of sequelae from chronic HCV infections ranged from $17,500 per year for nonadvanced fibrosis to $262,000 within the year after a liver transplant (*29*). Cost-benefit analyses show that universal opt-out screening in prisons is cost-effective, reducing ongoing HCV transmission, the incidence of advanced liver diseases, and death from liver disease (*24*). A 2020 study found that a test all, treat all, and linkage to care at release model would cost prisons $1,440 per person and result in a 23% increase in lifetime SVR and 54% reduction of cirrhosis cases (*30*).

## HCV Treatment in Carceral Settings—Real-World Examples

Financial and other barriers continue to limit access to HCV treatment in US carceral settings (Table 2). However, some initiatives have demonstrated promising outcomes.

**Table 2:** Real-world examples of direct-acting antiviral treatment in prisons and jails that advance hepatitis C elimination through opt-out universal screening and treatment in carceral settings, United States*

Reference	Institution	Years	Population	Policy	Outcome
(*53*)	Vermont Department of Corrections	2018–2020	HCV-infected patients (n = 217) in Vermont state prisons; 76% had opioid use disorder, 67% had a psychiatric comorbidity, and 9% had cirrhosis.	DAA treatment was initiated for all persons with positive HCV antibody and RNA tests.	A total of 217 (59%) persons started DAA treatment; of those, 129 (92%) completed treatment and 182 (84%) achieved documented SVR. Presence of psychiatric comorbidity and receipt of MOUD was not significantly associated with achieving SVR12.
(*51*)	NYC jails, services provided by Correctional Health Services	Jan 2014–Oct 2017	HCV-infected patients (n = 269) who were treated with DAA therapy while in NYC jail.	DAA treatment was initiated in all persons with sentence lengths greater than anticipated duration of therapy. Treatment was continued for all persons who were on DAAs in the community at the time of entry. A 7-day supply of medication was given to persons returning to the community before treatment completion.	269 persons, 88 (33%) persons continued DAA treatment started in the community and 118 (67%) persons started DAA treatment prescribed in jail. SVR data is available for 195 (72%) persons; of those, 172 (88%) achieved SVR12.

*DAA, direct-acting antiviral; HCV, hepatitis C virus; MOUD, medications for opioid use disorder (such as naltrexone or buprenorphine); NYC, New York, NY; SVR, sustained virologic response; SVR12, sustained virologic response 12 months after completing treatment (no detectable HCV RNA in blood).

### Innovative Payment Models

Despite recent cost reductions, DAA treatment remains expensive; an average wholesale price is $26,000–$90,000 per treatment course (*31*). Innovative payment models were launched by Louisiana and Washington in 2019 to reduce the cost of expensive medications. In Louisiana, DAAs purchased by Medicaid or the Department of Public Safety and Corrections count toward an expenditure cap, after which subsequent prescriptions receive rebates that have a nominal incremental cost. In Washington, a similar program was negotiated for Medicaid recipients. Washington also introduced a separate payment model where their DOC receives a discount off the wholesale acquisition cost of direct purchases, which does not have an expenditure cap. Although increased HCV treatment among Medicaid recipients has been shown in Washington and Louisiana, the effects of those innovative payment models on HCV treatment among incarcerated persons has not been reported (*32*).

### Decentralized HCV Care

The Extension for Community Healthcare Outcomes model, first piloted in New Mexico in 2003, uses telehealth consultations between HCV experts and on-site correctional health professionals to train primary care providers to treat hepatitis C (*33*). The model program also established a peer education program that trains incarcerated persons to educate their peers about risk factors for HCV infection, the consequences of infection, and benefits of treatment and enables persons who previously refused testing or treatment the opportunity to reconsider. The New Mexico Corrections Department began universal screening in 2018 and had a hepatitis C prevalence of 40%–45% in their carceral population (*34*). In 2020, New Mexico allocated $22 million over 5 years for hepatitis C testing and treatment; >2,100 persons were treated during 2021–2023 (*35*,*36*).

### Litigation and State Policy

Recent court rulings have shown that the threat of HCV-related litigation can expand access to treatment, expediting progress toward HCV elimination in carceral settings. Arguments primarily assert that denial of treatment violates the 8th Amendment of the US Constitution prohibiting cruel and unusual punishment (Table 3) (*37*,*38*). According to a seminal 1976 ruling in Estelle v. Gamble, carceral facilities must avoid deliberate indifference to patient health needs (*39*,*40*). Although AASLD/IDSA guidelines established universal hepatitis C treatment as a standard of care, carceral settings have used prioritization criteria to limit DAA treatment on the basis of liver fibrosis stage or other clinical manifestations. Courts have ruled differently on whether prioritization criteria used in some carceral settings constitute deliberate indifference (*41*).

**Table 3:** Litigation supporting HCV treatment of incarcerated persons that advances hepatitis C elimination through opt-out universal screening and treatment in carceral settings, United States*

Case	Court	Claims	Rulings
Estelle v. Gamble, 1976	US Supreme Court	Plaintiff was subjected to cruel and unusual punishment in violation of the 8th Amendment for inadequate treatment of a back injury sustained while he was engaged in prison work.	Judge ruled that correctional facilities cannot display deliberate indifference to known healthcare needs of incarcerated individuals.
Stafford v. Carter, 2018	US District Court, Indianapolis Division	98.8% of incarcerated people with chronic HCV infection were withheld DAAs per prison treatment allocation protocol, violating 8th Amendment to the US Constitution, the Americans with Disabilities Act, and the Rehabilitation Act.	Judge ruled that the prison's policy of relying on APRI scores to determine treatment eligibility amounted to deliberate indifference in this class action suit.
Postawko v. Missouri Department of Corrections, 2020	US District Court, Western District of Missouri, Central Division	Class action suit sought prospective relief for denial of rights endowed to plaintiffs by 8th Amendment to the US Constitution and the Americans with Disabilities Act, for systemic denial of treatment for individuals with chronic HCV infection.	Private settlement agreement to enforce universal opt-out screening at intake, perform reflex testing within 3 days of positive antibody result, invest $7 million annually to purchase DAAs and enforce treatment of all individuals at highest risk for complications or disease progression.

*Reflex testing describes the process by which the lab performs HCV antibody testing and, if reactive, uses the same sample to automatically perform HCV RNA testing. APRI, aspartate transaminase to platelet ratio Index; DAA, direct-acting antiviral; HCV, hepatitis C virus.

A federal class action suit representing persons with HCV infection who were denied treatment during incarceration was filed against the Tennessee DOC (Atkins v. Parker, 2016). In response to the lawsuit, the Tennessee legislature provided the Tennessee DOC with new funding for hepatitis C treatment ($25 million by 2019), even though the DOC’s prioritization policy was ultimately found to be lawful, and the ruling was affirmed on appeal (*42*). This investment increased the number of incarcerated persons receiving DAA treatment in the Tennessee DOC system from 1 in 2016 to 956 in 2021 (*43*).

## Intersection of HCV Elimination and Substance Use Treatment

Proponents of expanded DAA treatment to prevent chronic HCV infection in incarcerated persons must also contend with substance use disorder and the overdose crisis among persons who inject drugs (PWID). Although robust evidence exists indicating that providing sterile injecting equipment reduces HCV transmission, no carceral facility currently provides sterile injection equipment. Persons released from prisons or jails are 10–40 times more likely to die from an overdose than are persons in the general population; the greatest risk for death is 3–4 weeks after release (*44*,*45*). Medications for opioid use disorder (MOUD), such as methadone, naltrexone, and buprenorphine, are highly safe and efficacious. Exposure to MOUD during incarceration is associated with 85% reduction in all-cause mortality and 75% reduction in overdose-related deaths in persons after reentry into the community (*45*).

MOUD is a critical component of HCV prevention because it decreases unsafe injecting practices in PWID; MOUD alone can reduce the risk for HCV infection by 50% and reinfection by 73% among PWID (*46*). Initiating HCV treatment also increases the uptake of MOUD (*47*). As of 2022, a total of 15 laws across 12 US states had expanded access to MOUD in prisons and jails for substance use treatment (*45*).

## Linkage to Care

Lack of insurance coverage and lack of coordinated handoff between carceral and community healthcare systems complicate healthcare transitions for incarcerated persons after release (*48*). California was the first state to apply for Section 1115 waivers of the Medicaid Inmate Exclusion Policy to secure payment coverage for substance use treatment for persons who would otherwise lose coverage during incarceration under that Medicaid policy (*49*). Although DAAs are included, waivers are unlikely to directly increase DAA treatment because many persons are treated during incarceration. However, the waivers can substantially improve linkage to care for MOUD and mental health treatment to prevent new or recurrent HCV infection after release.

## Future Directions

National data on the incidence and prevalence of HCV in carceral settings is required to improve HCV surveillance efforts and monitor progress across the country. Collaboration between US public health organizations and DOCs is essential both for data collection and improved control of HCV transmission in the carceral setting. Identifying facility-specific barriers and allocating data-driven resources are critical to improve HCV surveillance and treatment.

The US Congress is currently considering funding for a National Hepatitis C Elimination Initiative. This $11.3 billion initiative would enhance screening, testing, treatment, prevention, and monitoring of hepatitis C for all Americans; the goal is to reach elimination targets within a 5-year period (*50*). Of note for incarcerated persons, the plan includes point-of-care HCV RNA testing, provider training and technical assistance for implementation, and a national drug procurement plan that would cover incarcerated or detained persons. Carceral facilities can make considerable steps toward elimination now by using universal opt-out screening and providing DAA and MOUD treatment, improving linkages to community care, and building data infrastructure to track progress toward HCV elimination.

Broadly implementing hepatitis C testing and treatment programs in US prisons and jails advances public health and health equity. HCV elimination in carceral settings not only profoundly affects a person’s health but also improves community health. Only through screening and treating hepatitis C in carceral health settings can we achieve national HCV elimination goals.
